# P03-12 Falling between two stools: parents' experience of supporting their children with cerebral palsy (GMFCS I-II) living an active life

**DOI:** 10.1093/eurpub/ckac095.048

**Published:** 2022-08-29

**Authors:** Sofie Morley, Christina Esmann Fonvig, Jens Troelsen, Anders Holsgaard Larsen

**Affiliations:** 1 Department of Sports Science and Clinical Biomechanics, University of Southern Denmark, Odense, Denmark; 2 The Orthopaedic Research Unit, Department of Clinical Research, University of Southern Denmark, Department of Orthopaedics and Traumatology, Odense University Hospital, Odense, Denmark

**Keywords:** cerebral palsy, physical activity, leisure activities, coaches

## Abstract

**Background:**

Children and adolescents with cerebral palsy (CP) are a vulnerable group who find it challenging to meet current physical activity guidelines, which predispose them to the negative health implications associated with low levels of physical activity and high levels of sedentary time. For these reasons, a key role for many clinicians, parents, and other practitioners is to encourage and facilitate an increase in habitual physical activity and reduce the amount of time spent sedentary, in order to optimize long-term health outcomes.

In Denmark, there is a strong tradition of practicing habitual exercise at leisure activities (83% of children and adolescents). When participating in leisure activities these children are being physically active as well as experiencing being a part of a community. Children and adolescents living with CP are often not able to participate in regular leisure activities, which excludes them from the active and social life that's happening there. This study focuses on parents of children with GMFCS I-II, who, due to their good walking abilities, may be more likely to be able to participate in regular leisure activities.

This study aims to explore the daily life and challenges described by parents in their pursuit of supporting their children with CP living a physically active life. This knowledge can be used by parents, clinicians, coaches, teachers, and other practitioners to guide families living with CP towards a more physically active lifestyle and possibly optimize long-term physical and social health outcomes for children and adolescents with CP.

**Methods:**

Eleven parents of children with CP (n = 7) (GMFCS I-II) aged 7-15 years participated in seven semi-structured interviews. Thematical analysis was performed to understand the data material.

**Results and conclusions:**

The main challenge described by parents was formulated as ‘Falling between two stools’ referring to the experience of their child being ‘too good’ for adapted leisure activities, but at the same time wanting to participate in regular leisure activities, but performing a little too poorly, to feel included. It was found that a reigning performance culture in regular leisure activities was perceived as a barrier for the participation of these children.

